# A novel self-expanding biflanged metal stent vs tubular metal stent for EUS-guided transmural drainage of pancreatic pseudocyst: A retrospective, cohort study

**DOI:** 10.1097/MD.0000000000014179

**Published:** 2019-01-18

**Authors:** Yao Yao, Dingguo Zhang, Jiefang Guo, Ke Qi, Feng Li, Jianwei Zhu, Dong Wang, Jie Chen, Can Xu, Luowei Wang, Kaixuan Wang, Zhendong Jin, Zhaoshen Li

**Affiliations:** a Department of Gastroenterology, Changhai Hospital, Second Military Medical University; b Department of Gastroenterology, Shenzhen People's Hospital, Second Clinical Medical College of Jinan University, Shenzhen, China; c Department of Gastroenterology and Hepatology, CHRISTUS Mother Frances Hospital, Tyler, TX.

**Keywords:** drainage, endoscopic ultrasound, pancreatic pseudocyst, stent

## Abstract

Although endoscopic ultrasound (EUS)-guided transmural drainage of pancreatic fluid collections with metal stents is generally preferred over plastic stents, its superiority among different types of metal stents has not yet been well studied. We conducted this study to compare clinical outcomes and complications of a novel self-expanding biflanged metal stent (BFMS) and a traditional-shaped tubular metal stent (TMS) in treating pancreatic pseudocyst (PPC).

This was a retrospective analysis on consecutive patients with PPC underwent EUS-guided transmural drainage with either TMS or BFMS in a single tertiary center with expertise in management of complex biliary and pancreatic problems. The technical and functional success rate, reintervention, complications, and recurrence rate were evaluated.

From September 2013 to January 2018, 125 patients (66.4% male, median age 47 years) underwent EUS-guided transmural drainage for PPC. Among them, 49 used TMS and 76 used BFMS. All patients met the inclusion criteria that cyst diameter was >6 cm or the distance between cyst and stomach wall was shorter than 1 cm. There was no difference in technical success (98% vs 97.4%, *P* = 1.0) or functional success rate (87.8% vs 92.1%, *P* = .54) using 2 types of metal stents. However, more procedure related complications occurred in TMS than in BFMS group. TMS group had a much higher migration rate than BFMS group (14.6% vs 0, *P* = .001), even though there was no significant difference in bleeding, infection, or death rate between 2 groups. With similar clinical outcomes, TMS group required more additional plastic stent placement than BFMS group for better drainage.

TMS and BFMS placement can both be considered as methods of endoscopic transmural PPC drainage with equal efficacy, whereas BFMS could be preferred for fewer complications or less need of additional plastic stent placement.

## 1 Introduction

Endoscopic transmural drainage is a minimally invasive alternative to surgery for drainage of pancreatic pseudocyst (PPC).^[1,2]^ There are several multicenter studies on stent selection associated with successful endoscopic ultrasound (EUS)-guided drainage of PPC.^[3,4]^ Current clinical researches indicated that using self-expanding metal stents (SEMSs) for EUS-guided drainage may be able to treat PPC more efficiently, safely, and consequently yield better treatment outcome than using plastic stents.^[5,6]^ The main advantage of a SEMS is its larger luminal diameter (≥10 mm), which potentially results in longer stent patency, faster, and more sufficient PPC resolution, a reduced need for endoscopic reintervention, and a lasting access route for necrosectomy.

The use of different types of SEMSs has been reported in case reports and small case series recently. Most of these SEMSs were tubular stents which were initially designed for bile duct drainage.^[7–9]^ Even though the technical success rates (TSRs) were reported as high as 89% to 100%, the clinical treatment success in PPC with tubular full-covered metal stent was not always guaranteed (78–95%). High risk (15–44% in large case series) of complications like infection, perforation, migration, and bleeding were reported.^[10–13]^ It is indicated that quite a number of patients using tubular metal stent (TMS) required additional interventions to avoid procedure-related complications.^[14,15]^

In the recent 5 years, several studies reported on the use of self-expanding biflanged metal stent (BFMS) with high TSRs (91–100%) and functional success rates (FSRs; 81–100%).^[10,16,17]^ Reports on BFMS claimed that EUS-guided drainage using the stent is feasible and the clinical results obtained are promising with a low major complication rate. However, 6–17% of complications were still reported.^[13,18,19]^ In addition, previous studies on evaluation of metal stent drainage mostly were of small scale and lack of control or comparison.^[20,21]^ Thus, the advantages of BFMS over other metal stents for the treatment of PPC stay unclear and the criteria for PPC that are best treated with BFMS remain to be determined. To meet requirements for better drainage efficiency and fewer complications, a novel fully covered, self-expanding BFMS with anchoring flanges at both ends, has been developed by Micro-Tech Co Ltd (Nanjing, China) specifically for EUS-guided transmural drainage of PPC.

Until now, no head to head comparative studies on the safety and efficacy of TMS and BFMS have been reported yet. Which type of metal stent placement is better during EUS-guided transmural drainage for PPC? The present study sought to provide some insight for selection of transmural metal stents during EUS-guided drainage of PPC by comparing the technical and clinical outcomes and complications of this novel BFMS with TMS.

## 2 Materials and methods

### 2.1 Participants

The study has been approved by the institutional review board of Shanghai Hospital. From September 2013 to January 2018, all patients diagnosed with PPC, who underwent EUS-guided transmural metal stent drainage in our hospital, were retrospectively identified and reviewed in a web-based database. PPCs were identified based on history of the patients and findings on EUS, computed tomography (CT), or magnetic resonance imaging, as defined by the revised 2012 Atlanta classification.^[22]^ Patients whose maximum diameters of the pseudocysts were <6 cm or the distance between pseudocyst and stomach wall was >1 cm were excluded. More detailed selection criteria are shown in Table 1. The decision to place a metal stent and selection of stent type were at the discretion of experienced advanced endoscopists who performed the transmural drainage. Written informed consent was obtained from all patients prior to the procedure of stent placement.

**Table 1:**
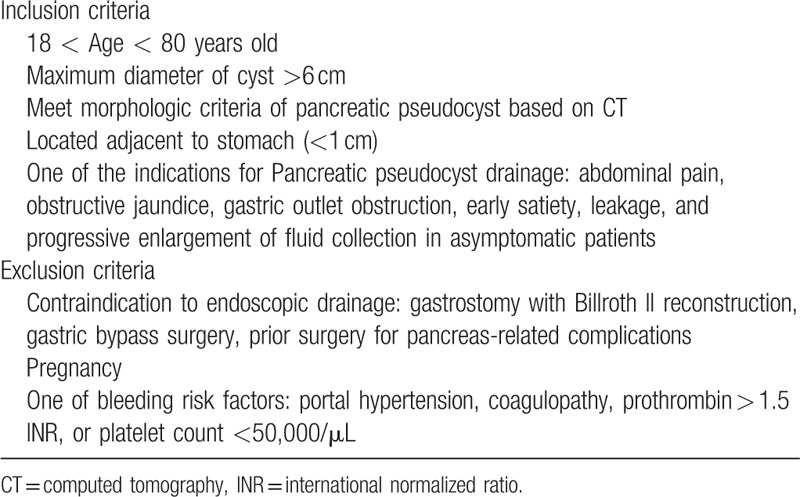
Inclusion and exclusion criteria.

### 2.2 Stents

**Figure 1:**
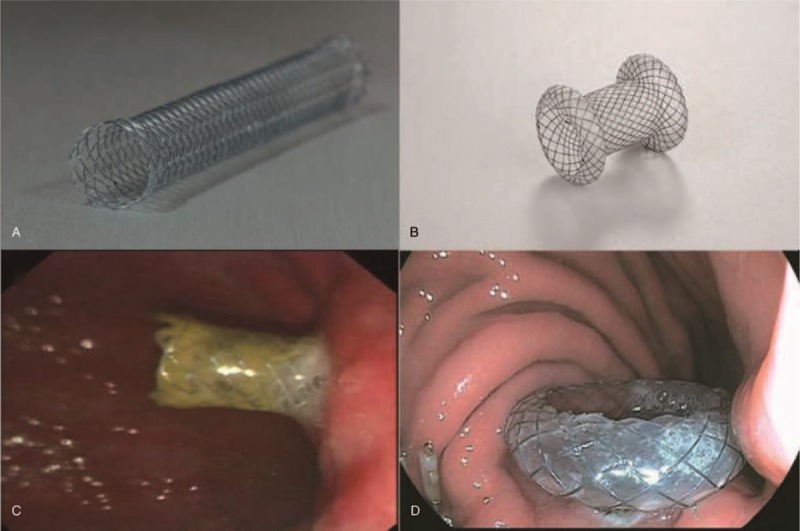
Endoscopic ultrasound-guided transmural placement of self-expanding tubular metal stent (A, C) and biflanged metal stent (B, D) for drainage of pancreatic pseudocyst.

Fully covered 10 mm × 60 mm TMS (ST03-003.10.060; Micro-Tech Co Ltd) and novel fully covered BFMS, 16 mm diameter × 30 mm length in body, 20 mm diameter×5 mm length in the flanged end (ST33-103.16.30; Micro-Tech Co Ltd) were used in this study (Fig. 1A, B). The BFMS design, with wide flanges on both ends, provides anchoring flanges within the PPC and an even distribution of pressure on the luminal walls. The metal material of these silicon-dioxide-coated stents is Nitinol memory alloy.

### 2.3 Procedure

All procedures were performed under linear array echoendoscope (GF-UCT180; Olympus) guidance at the discretion of endoscopist while the patients were under general anesthesia with endotracheal intubation. EUS was performed to locate the PPC, measure and confirm the size, and assess the fluid content.

Under EUS guidance, the collection was punctured from the stomach with a 19-gauge EUS-guided fine-needle aspiration needle (ECHO-19; Cook Medical). A 0.035-inch guidewire (Jagwire; Boston Scientific) was passed through the needle and was coiled in the PPC. After needle withdrawal, a 10-Fr cystotome needle knife (G30550; Cook Medical) was placed over the guidewire and inserted into the cavity. The cystotome was then removed and the stent (TMS or BFMS) was deployed in the PPC lumen under fluoroscopic and endoscopic guidance (Fig. 1C, D).

### 2.4 Management during follow-up evaluation

All patients who underwent procedures were transferred to their rooms and discharged when their symptoms improved. If patients had inadequate symptom relief or presented with new-onset abdominal pain, a CT of the abdomen was performed to assess treatment response. If there was evidence of inadequate drainage or stent occlusion, a nasocystic catheter placement and lavage of the internal cavity by intermittent irrigation with 500 to 1000 mL of normal saline solution would be performed. If inflammation continued, additional irrigation or additional plastic stent placement was conducted. Direct endoscopic necrosectomy (DEN) was performed the following day. Necrotic tissue was removed using stone retrieval baskets, biopsy forceps, and snare forceps with CO_2_ insufflation.

The end point of DEN was the relief of symptoms and systemic inflammatory response syndrome. Transmural stents were removed if the PPC had resolved for 2 months on CT scan which was performed routinely in all patients. In patients with persistent or recurrent symptoms, imaging studies were repeated when necessary. In patients with persistent or recurrent PPC for more than 2 months, further treatment was performed by either a repeat intervention or the patient was crossed over to the alternate treatment arm.

### 2.5 Outcome measures and definitions

The primary outcome measures included TSR and FSR. The secondary outcome measures were complications and median time to pseudocyst recurrence.

Pseudocyst located near the head or the uncinate region of pancreas was classified as being in the head. Pseudocyst located adjacent to the body of the pancreas or extending to the body–tail junction (from the body of the pancreas) was classified as being in the body. PPC localized to the tail of the pancreas or extending to the body–tail junction (from the tail) was classified as in the tail.^[23]^

The TSR was defined as satisfactory access and drainage of the PPC following placement of the metal stent. FSR was defined as complete resolution or a decrease in size of the fluid collection to 2 cm or smaller on CT in association with resolution of symptoms at the 8-week outpatient follow-up evaluation.

Safety was measured by complications (including bleeding, infection, peritonitis, and perforation). Bleeding was defined as any hemorrhagic event ring during or after the procedure that required any intervention or blood transfusion. Suspicion of infection is usually based on ongoing clinical deterioration despite maximal medical support, body temperature above 38.0°C for more than 48 hours with white blood cell count rising above 10^10^ mmol/L and positive blood cultures after the initial endoscopic drainage. Peritonitis referred to consistent abdominal pain which is usually caused by ductal leakage. Stent migration was defined that if an intervention was required to retrieve the stent either from within the PPC or from the enteral lumen during follow-up. Recurrence referred to a return of symptoms and lesions at the same site in pancreas within 6-month treatment.

### 2.6 Statistical analysis

Statistical analysis was performed using SPSS 17.0 (IBM SPSS Software) and a 2-tailed *P*-value of <.05 was considered statistically significant. Variables for age, size of pseudocysts, and follow-up in the 2 groups were analyzed using the Student *t* test or Mann–Whitney *U* test, respectively, for continuous data with normal or nonnormal distributions. Differences in categorical variables for technical success, complications, and treatment outcome measures were analyzed using the Chi-squared and Fisher exact test.

## 3 Results

### 3.1 Technical and functional success

Between September 2013 and January 2018, a total of 125 patients undergoing EUS-guided drainage for PPC using TMSs (n = 49) or BFMS (n = 76) were included in this study. The 2 groups had similar demographic characteristics, symptoms, locations, and sizes of PPC (Table 2).

**Table 2:**
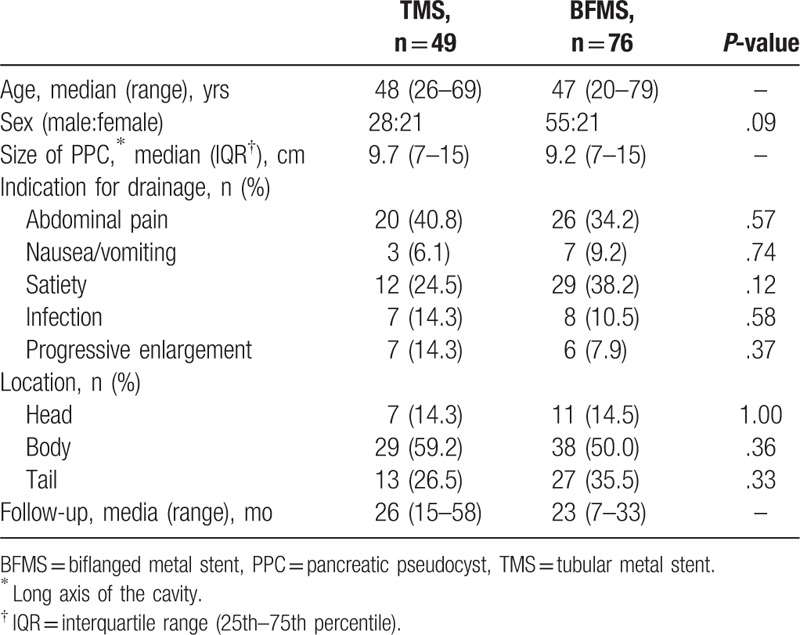
Demographic and clinical data.

There were no statistically significant differences between TMS and BFMS groups in terms of TSR (48/49, 98% vs 74/76, 97.4%, *P* = 1.0). One patient from BFMS group and the 1 from TMS group failed to place metal stents because of interference of surrounding blood vessels. Another patient from BFMS group had an accidentally stent falling into the cyst cavity during the procedure and the patient had immediate surgical removal of the stent (Table 3).

**Table 3:**
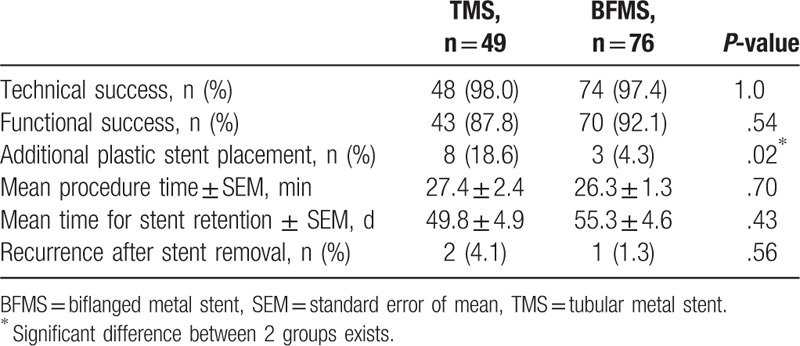
Comparison of clinical outcomes between 2 groups.

The FSR between TMS and BFMS groups were 43/49 (87.8%) and 70/76 (92.1%, *P* = .54). Six patients of TMS group did not achieve functional success within 8 weeks follow-up. Three of them developed cavity enlargement and pancreatic abscess. The other 3 patients developed severe intrapseudocyst hemorrhage. There were also 6 patients of BFMS group who failed to achieve functional success. Three of them developed pancreatic abscess, 2 developed intrapseudocyst hemorrhage, and 1 patient did not gain symptoms relief (Table 3).

There are more patients in TMS group who need additional plastic stent placement for better drainage than BFMS group (8/49, 18.6% vs 3/76, 4.3%, *P* = .02). More than 50% of patients achieved complete resolution within 2 weeks after procedure. There was no significant difference between TMS and BFMS groups in procedure time (27.4 ± 2.4 vs 26.3 ± 1.3 minutes, *P* = .70) (Table 3).

### 3.2 Complications

There were 1 patient in TMS group and 1 in BFMS group who developed peritonitis, which was presumably due to pancreatic juice leakage (Table 4). Both patients recovered completely with supportive medical therapy.

**Table 4:**
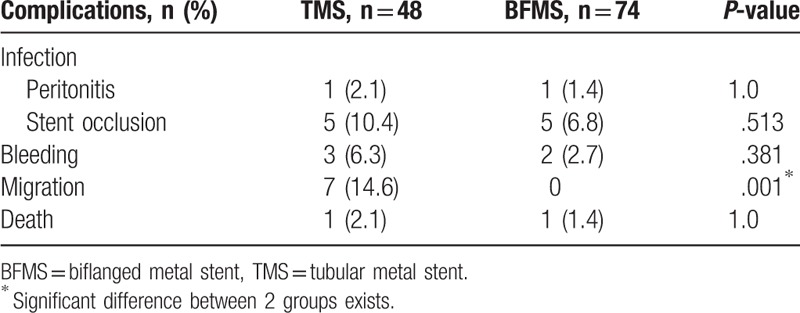
Comparison of procedure related complications between 2 groups.

The patients who had infection caused by stent occlusion received antibiotic therapy and either nasocystic catheter or additional plastic stent drainage, and finally recovered. Repeated DENs were also performed to unblock the drainage channel of the stent.

Bleeding occurred in 3 patients in TMS group and 2 patients in BFMS group. Attempts of endoscopic hemostasis were not successful, emergency interventional radiologic embolization was performed, and 1 patient of TMS group achieved temporary hemostasis. The other 2 patients in TMS group had surgeries after coil embolization failed to achieve hemostasis. However, 1 patient died. One in BFMS group achieved hemostasis by surgery but the other 1 died of severe hemorrhage despite maximum rescue including interventional embolization and surgery (Table 4).

Intraluminal stent migrations were identified by follow-up abdominal radiograph. Endoscopic stent removal was not performed due to spontaneous stent passage. On further imaging (abdominal radiograph, abdominal ultrasound, or upper gastrointestinal endoscopy), these stents were no longer visualized. None of these patients experienced symptoms due to intraluminal stent migration.

In BFMS group, hyperplastic tissue ingrowth of the stent occurred in 2 patients, but stent removal was uneventful with the discretion of the endoscopists.

By comparison, there was no significant difference in infection (6/48, 12.5% vs 6/74, 8.2%, *P* > .05) and bleeding rate (3/48, 6.3% vs 2/74, 2.7%, *P* > .05) between TMS and BFMS groups. However, we found that stent migration (14.6% vs 0%, *P* = .001) occurred more frequently in TMS than BFMS group (Table 4).

### 3.3 Follow-up and recurrence rate

The average time for stent retention is 49.8 ± 4.9 days for TMS group and 55.3 ± 4.6 days for BFMS group. Patients in TMS and BFMS groups were followed up after stent retrieval for a median time of 26 months (range 15–58 months) and 23 months (range 7–33 months), respectively (Table 2). Two patients in TMS group had pseudocyst recurrence in 4th and 11th month after stent removal, respectively, with suspected disrupted pancreatic duct. Both of them were treated with endoscopic retrograde cholangiopancreatography (ERCP) and nasocystic catheter drainage successfully. One patient of BFMS group had recurrence in 9th month after stent placement. He was treated with another EUS-guided transmural BFMS placement for drainage (Table 3).

## 4 Discussion

Traditional tubular biliary metal stent and BFMS have been widely used for EUS-guided drainage of PPC in recent years, but most of these studies were usually restricted to small case series and observation reports.^[14,20,24]^ To the best of our knowledge, there has been only 1 comparative study showing distinctions among different types of metal and plastic stents for PPC drainage.^[25]^

Successful insertion of metal stents in both groups turned out to be higher than 97%, which is consistent with the available medical literature.^[10,24–26]^ This mainly attributed to verifying the presence of a collection as seen on CT or EUS guidance before drainage, which showed the optimal site for drainage, ensuring a minimal distance and avoiding vessel interposition. In terms of FSR, TMS and BFMS for PPC drainage were 87.8% and 92.1%, respectively, in present study, which was in accordance with the existing medical literature: 77.8% to 100%.^[24,27–29]^

Several studies on the use of SEMSs for EUS-guided drainage showed that PPC infections were usually caused by recurrent stent occlusion by necrotic debris.^[24,30]^ DEN or nasocystic catheter drainage can help drain necrosis from the cavity and prevent obstruction of the stent outlet in stomach.^[5,31]^ In our study, we found no significant difference in transient infection rate between TMS and BFMS groups. DEN and nasocystic catheter drainage were performed successfully to deal with stent occlusion. It is mentioned that we used necrosectomy to remove necrotic tissues which may have caused procedure-related inflammation. But we do not think these cases can be diagnosed as walled off pancreatic necrosis (WOPN) at the beginning. The necrotic tissues may be related to complications. There were 5 cases of bleeding occurred in this study, including 3 in TMS group and 2 in BFMS group. As a previous study suggested, this may be explained by sharp ends of TMS with eroding into the gastric mucosa or retroperitoneum causing ulceration and bleeding. Bapaye et al reported one case of hemorrhage and explained that partial erosion of the inner end of the stent into the wall of the splenic artery causing a pseudo-aneurysm and extravasation of contrast from the cavity into the stomach.^[28]^ Rinninella et al claimed massive bleeding was caused by nasocystic drainage catheter.^[12]^ However, Siddiqui et al reported that lumen-apposing metal stent (LAMS) with wide flanged at both ends was more likely to have bleeding, which was explained by stent erosion into a vessel as the cavity wall collapses.^[25]^ But in this study, less bleeding was observed in BFMS group, may be partly because of the BFMS we used have much smaller diameter and shorter length.

Theoretically, the stent migration rate of BFMS group was lower than TMS group because the anchoring flanges at both ends of BFMS can keep the stent from moving. And it was demonstrated that tissue ingrowth/overgrowth at both stent ends also helped reduce migration.^[7,32]^ Despite the anchoring design on BFMS, stent migration rate has been reported from 5% to 7% in several studies. Previous observations reported that rapid cyst decompression with SEMS may be a key factor in the etiology of spontaneous migration that overcomes the flared ends.^[5,10,32,33]^

Stents falling into cyst cavity during the procedure occurred in 3 cases, which was primarily due to lack of experience with the new BFMS system. The stents were pulled out of the cysts and relocated immediately.

Hyperplastic tissue ingrowth of the stent was observed in 2 cases in BFMS group, which brought difficulties in the removing process. We agree with a previous study that the duration of the stent placement resulted in loss of the stent coating because these stents were in situ for 4 to 5 months.^[24]^ Barresi et al reported that 1 patient received surgery to remove a stent that became fully embedded in the stomach wall.^[34]^ The occurrence of tissue ingrowth across our study indicated that BFMS may not be suitable for management of PPC which require long-time stent retention.

Few studies reported recurrence rate for small number of cases or lack of long-term follow-up data. Three cases of recurrence in TMS group were probably related to pancreatic duct disruption, which was in accordance with a prospective study.^[20]^

One potential advantage of this new BFMS system is its large diameter (16 mm). It would allow therapeutic endoscopic maneuvers such as necrosectomy, gastrojejunostomy stenting, BFMS-assisted ERCP in gastric bypass patients, etc.

There are some strengths and limitations to this study. Firstly, this is the 1st retrospective cohort study on comparison of 2 types of metal stents for EUS-guided drainage of PPC published to date. Secondly, 7 to 58 months follow-up was carried out and recurrence rate was evaluated in our report, which was absent in majority of previous studies.

However, the study design was retrospective with its inherent limitations. Cost analysis was not included in our study, either. Furthermore, selection bias may exist because stent selection was up to the decisions of experienced endoscopists. Now some authors prefer application of 2 double pig tail plastic stents while metal stents were left for WOPN as metal stent has more incidence of serious cyst bleeding. It is true that we compared complications between 2 kinds of metal stents without additional plastic stents, which appears to be the limitation of this study. Maybe in the future we can put more groups into this study.

We also think it will be very beneficial to compare this novel BFMS with the LAMS as Nagi stents and hot axios stents. LAMSs were also used in several patients at our hospital recently. The number of patients is too small and follow-up is not long enough. We will keep following and enlarge patients number so that we may make another compare in the future.

## 5 Conclusion

This single center retrospective cohort study made a comparison of 2 types of SEMSs for EUS-guided drainage of PPC. We demonstrated that both TMS and BFMS drainage were feasible, with high technical and FSR for PPC. Due to the anchoring flanges at both ends of BFMS which can help keep the stent from moving, the clinical outcomes of BFMS obtained are more promising with a lower migration rate than TMS. And larger diameter of BFMS may result in less need of additional plastic stent placement for better drainage. However, due to the limitations of our study, future larger randomized, and prospective trials evaluating the role of metal stents placement in transmural PPC drainage are needed.

## Author contributions

**Conceptualization:** Dingguo Zhang, Kaixuan Wang, Zhendong Jin.

**Data curation:** Yao Yao, Jiefang Guo, Jianwei Zhu.

**Formal analysis:** Jiefang Guo, Jianwei Zhu.

**Funding acquisition:** Dingguo Zhang, Kaixuan Wang.

**Investigation:** Jiefang Guo.

**Methodology:** Dingguo Zhang, Dong Wang.

**Project administration:** Dong Wang, Jie Chen, Can Xu, Luowei Wang.

**Resources:** Ke Qi, Zhaoshen Li.

**Software:** Ke Qi.

**Supervision:** Kaixuan Wang.

**Validation:** Feng Li, Zhendong Jin.

**Visualization:** Zhendong Jin.

**Writing – original draft:** Yao Yao.

**Writing – review & editing:** Feng Li, Kaixuan Wang, Zhaoshen Li.

## References

[1] Varadarajulu S Bang JY Sutton BS Equal efficacy of endoscopic and surgical cystogastrostomy for pancreatic pseudocyst drainage in a randomized trial. Gastroenterology 2013;145:583–90. 23732774 10.1053/j.gastro.2013.05.046

[2] Patil R Ona MA Papafragkakis C Endoscopic ultrasound-guided placement of AXIOS stent for drainage of pancreatic fluid collections. Ann Gastroenterol 2016;29:168–73. 27065729 10.20524/aog.2016.0008 PMC4805736

[3] Lin H Zhan XB Sun SY Stent selection for endoscopic ultrasound-guided drainage of pancreatic fluid collections: a multicenter study in China. Gastroenterol Res Pract 2014;2014:193562. 25018767 10.1155/2014/193562 PMC4074944

[4] Bang JY Varadarajulu S Metal versus plastic stent for transmural drainage of pancreatic fluid collections. Clin Endosc 2013;46:500–2. 24143311 10.5946/ce.2013.46.5.500 PMC3797934

[5] Walter D Will U Sanchez-Yague A A novel lumen-apposing metal stent for endoscopic ultrasound-guided drainage of pancreatic fluid collections: a prospective cohort study. Endoscopy 2015;47:63–7. 25268308 10.1055/s-0034-1378113

[6] Mukai S Itoi T Baron TH Endoscopic ultrasound-guided placement of plastic vs. biflanged metal stents for therapy of walled-off necrosis: a retrospective single-center series. Endoscopy 2015;47:47–55. 25264765 10.1055/s-0034-1377966

[7] Penn DE Draganov PV Wagh MS Prospective evaluation of the use of fully covered self-expanding metal stents for EUS-guided transmural drainage of pancreatic pseudocysts. Gastrointest Endosc 2012;76:679–84. 22732874 10.1016/j.gie.2012.04.457

[8] Fabbri C Luigiano C Cennamo V Endoscopic ultrasound-guided transmural drainage of infected pancreatic fluid collections with placement of covered self-expanding metal stents: a case series. Endoscopy 2012;44:429–33. 22382852 10.1055/s-0031-1291624

[9] Sarkaria S Sethi A Rondon C Pancreatic necrosectomy using covered esophageal stents: a novel approach. J Clin Gastroenterol 2014;48:145–52. 23751853 10.1097/MCG.0b013e3182972219

[10] Itoi T Binmoeller KF Shah J Clinical evaluation of a novel lumen-apposing metal stent for endosonography-guided pancreatic pseudocyst and gallbladder drainage (with videos). Gastrointest Endosc 2012;75:870–6. 22301347 10.1016/j.gie.2011.10.020

[11] Lee BU Song TJ Lee SS Newly designed, fully covered metal stents for endoscopic ultrasound (EUS)-guided transmural drainage of peripancreatic fluid collections: a prospective randomized study. Endoscopy 2014;46:1078–84. 25412095 10.1055/s-0034-1390871

[12] Rinninella E Kunda R Dollhopf M EUS-guided drainage of pancreatic fluid collections using a novel lumen-apposing metal stent on an electrocautery-enhanced delivery system: a large retrospective study (with video). Gastrointest Endosc 2015;82:1039–46. 26014960 10.1016/j.gie.2015.04.006

[13] Shah RJ Shah JN Waxman I Safety and efficacy of endoscopic ultrasound-guided drainage of pancreatic fluid collections with lumen-apposing covered self-expanding metal stents. Clin Gastroenterol Hepatol 2015;13:747–52. 25290534 10.1016/j.cgh.2014.09.047

[14] Chaves DM Mönkemüller K Carneiro F Endoscopic treatment of large pancreatic fluid collections (PFC) using self-expanding metallic stents (SEMS) - a two-center experience. Endosc Int Open 2014;2:E224–9. 26135097 10.1055/s-0034-1390796 PMC4423292

[15] Guo J Feng L Sun S Risk factors for infection after endoscopic ultrasonography-guided drainage of specific types of pancreatic and peripancreatic fluid collections (with video). Surg Endosc 2016;30:3114–20. 26801793 10.1007/s00464-015-4557-3 PMC4912585

[16] Bapaye A Dubale NA Sheth KA Endoscopic ultrasonography-guided transmural drainage of walled-off pancreaticnecrosis: Comparison between a specially designed fully covered bi-flanged metalstent and multiple plastic stents. Dig Endosc 2017;29:104–10. 27463528 10.1111/den.12704

[17] Siddiqui AA Adler DG Nieto J EUS-guided drainage of peripancreatic fluid collections and necrosis by using a novel lumen-apposing stent: a large retrospective, multicenter U.S. experience (with videos). Gastrointest Endosc 2016;83:699–707. 26515956 10.1016/j.gie.2015.10.020

[18] Bang JY Hasan MK Navaneethan U Lumen-apposing metal stents for drainage of pancreatic fluid collections: when and for whom? Dig Endosc 2017;29:83–90. 27199157 10.1111/den.12681

[19] Bang JY Hasan M Navaneethan U Lumen-apposing metal stents (LAMS) for pancreatic fluid collection (PFC) drainage: may not be business as usual. Gut 2017;66:2054–6. 27582509 10.1136/gutjnl-2016-312812 PMC5749339

[20] Gornals JB Consiglieri CF Busquets J Endoscopic necrosectomy of walled-off pancreatic necrosis using a lumen-apposing metal stent and irrigation technique. Surg Endosc 2016;30:2592–602. 26335077 10.1007/s00464-015-4505-2

[21] Huggett MT Oppong KW Pereira SP Endoscopic drainage of walled-off pancreatic necrosis using a novel self-expanding metal stent. Endoscopy 2015;47:929–32. 26126156 10.1055/s-0034-1392413

[22] Banks PA Bollen TL Dervenis C Classification of acute pancreatitis--2012: revision of the Atlanta classification and definitions by international consensus. Gut 2013;62:102–11. 23100216 10.1136/gutjnl-2012-302779

[23] Park DH Lee SS Moon SH Endoscopic ultrasound-guided versus conventional transmural drainage for pancreatic pseudocysts: a prospective randomized trial. Endoscopy 2009;41:842–8. 19798610 10.1055/s-0029-1215133

[24] Rinninella E Kunda R Dollhopf M EUS-guided drainage of pancreatic fluid collections using a novel lumen-apposingmetal stent on an electrocautery-enhanced delivery system: a large retrospectivestudy (with video). Gastrointest Endosc 2015;82:1039–46. 26014960 10.1016/j.gie.2015.04.006

[25] Siddiqui AA Kowalski TE Loren DE Fully covered self-expanding metal stents versus lumen-apposing fully covered self-expanding metal stent versus plastic stents for endoscopic drainage of pancreatic walled-off necrosis: clinical outcomes and success. Gastrointest Endosc 2017;85:758–65. 27566053 10.1016/j.gie.2016.08.014

[26] Yamamoto N Isayama H Kawakami H Preliminary report on a new, fully covered, metal stent designed for the treatment of pancreatic fluid collections. Gastrointest Endosc 2013;77:809–14. 23453183 10.1016/j.gie.2013.01.009

[27] Gornals JB De la Serna-Higuera C Sánchez-Yague A Endosonography-guided drainage of pancreatic fluid collections with a novel lumen-apposing stent. Surg Endosc 2013;27:1428–34. 23232994 10.1007/s00464-012-2591-y

[28] Bapaye A Itoi T Kongkam P New fully covered large-bore wide-flare removable metal stent for drainage of pancreatic fluid collections: results of a multicenter study. Dig Endosc 2015;27:499–504. 25545957 10.1111/den.12421

[29] Singhal S Rotman SR Gaidhane M Pancreatic fluid collection drainage by endoscopic ultrasound: an update. Clin Endosc 2013;46:506–14. 24143313 10.5946/ce.2013.46.5.506 PMC3797936

[30] Weilert F Binmoeller KF Shah JN Endoscopic ultrasound-guided drainage of pancreatic fluid collections with indeterminate adherence using temporary covered metal stents. Endoscopy 2012;44:780–3. 22791588 10.1055/s-0032-1309839

[31] Capone P Petrone MC Dabizzi E Endoscopic ultrasound-guided drainage of a pancreatic fluid collection using a novel lumen-apposing metal stent complicated by stent occlusion. Endoscopy 2016;48 Suppl 1:E203. 27285655 10.1055/s-0042-108572

[32] Talreja JP Shami VM Ku J Transenteric drainage of pancreatic-fluid collections with fully covered self-expanding metallic stents (with video). Gastrointest Endosc 2008;68:1199–203. 19028232 10.1016/j.gie.2008.06.015

[33] Kahaleh M Tokar J Conaway MR Efficacy and complications of covered Wallstents in malignant distal biliary obstruction. Gastrointest Endosc 2005;61:528–33. 15812404 10.1016/s0016-5107(04)02593-3

[34] Barresi L Tarantino I Curcio G Buried stent: new complication of pseudocyst drainage with self-expandable metallic stent. Dig Endosc 2012;24:285. 22725121 10.1111/j.1443-1661.2011.01205.x

